# Prevalence of COVID-19 IgG Antibodies in a Cohort of Municipal First Responders

**DOI:** 10.1017/S1049023X2000151X

**Published:** 2021-01-05

**Authors:** Sarayna S. McGuire, Aaron B. Klassen, John Heywood, Matthew D. Sztajnkrycer

**Affiliations:** 1. Department of Emergency Medicine, Mayo Clinic, Rochester, Minnesota USA; 2. Division of Prehospital Care, Mayo Clinic, Rochester, Minnesota USA; 3. Mayo Clinic Labs, Mayo Clinic, Rochester, Minnesota USA

**Keywords:** COVID-19, first responders, SARS-CoV-2, serology, testing

## Abstract

**Background:**

Although first responders (FRs) represent a high-risk group for exposure, little information is available regarding their risk of coronavirus disease 2019 (COVID-19) infection. The purpose of the current study was to determine the serological prevalence of past COVID-19 infection in a cohort of municipal law enforcement (LE) and firefighters (FFs).

**Methods:**

Descriptive analysis of a de-identified data reporting Severe Acute Respiratory Syndrome Coronavirus-2 (SARS-CoV-2) immunoglobulin G (IgG), or COR2G, serology results for municipal FRs. As part of the serology process, FRs were surveyed for COVID-19-like symptoms since February 2020 and asked to report any prior COVID-19 nasal swab testing. Descriptive statistics and two-sided Chi Square tests with Yates correction were used to compare groups.

**Results:**

Of 318 FRs, 225 (80.2%) underwent serology testing (LE: 163/207 [78.7%]; FF: 92/111 [82.9%]). The prevalence of positive serology for all FRs tested was 3/255 (1.2%). Two LE (1.2%) and one FF (1.1%) had positive serology (P = 1.0). Two hundred and twenty-four FRs responded to a survey regarding prior symptoms and testing. Fifty-eight (25.9%) FRs (44 LE; 14 FFs) reported the presence of COVID-19-like symptoms. Of these, only nine (15.5%) received reverse transcriptase – polymerase chain reaction (RT-PCR) testing; none were positive. Two of the three FRs with positive serology reported no COVID-19-like symptoms and none of these responders had received prior nasal RT-PCR swabs. The overall community positive RT-PCR rate was 0.36%, representing a three-fold higher rate of positive seroprevalence amongst FRs compared with the general population (P = .07).

**Conclusions:**

Amongst a cohort of municipal FRs with low community COVID-19 prevalence, the seroprevalence of SARS-CoV-19 IgG Ab was three-fold greater than the general community. Two-thirds of positive FRs reported a lack of symptoms. Only 15.5% of FRs with COVID-19-like symptoms received RT-PCR testing. In addition to workplace control measures, increased testing availability to FRs is critical in limiting infection spread and ensuring response capability.

## Introduction

As of September 6, 2020, more than 26.9 million confirmed cases of infection by Severe Acute Respiratory Syndrome Coronavirus-2 (SARS-CoV-2), the etiologic agent of coronavirus disease 2019 (COVID-19), have been reported world-wide, with 6.24 million cases reported in the United States alone.^1^ The US national employment data indicate individuals employed in the Protective Service Occupation, including first responders (FRs), are at a significant risk for infections such as COVID-19, with 52% reporting exposure to diseases or infections more than once a month.^2^ Efforts to control COVID-19 spread among FRs have largely emphasized workplace control measures, including ensuring access to and use of personal protective equipment (PPE), handwashing, physical distancing, workplace restrictions, environmental cleaning, and stay-at-home orders for those who have been exposed and remain asymptomatic.^3^

Rapid sequencing of the viral genome has allowed for early development of nucleic-acid-based polymerase chain reaction (PCR) tests that have been widely used to diagnose acute infections. Serology testing has emerged as an adjunct to PCR testing, particularly in terms of determining community prevalence. A recent study in China evaluating serology of 173 positive COVID-19 cases found the seroconversion rate for total antibody (Ab), immunoglobulin M (IgM), and immunoglobulin G (IgG) was 100.0%, 94.3%, and 79.8% at 15 days post-symptom-onset, respectively.^4^

Although FRs represent a high-risk group for exposure, little information is available regarding the risk of COVID-19 infection amongst FRs. As of August 3, 2020, 35 Emergency Medical Service personnel in the United States are reported to have died from COVID-19.^5^ A study of health care workers demonstrated that they accounted for 11% of COVID-19 infections.^6^ Limited data available from the 2003 SARS epidemic in one metropolitan area in Taiwan demonstrated an incidence of probable SARS infection among emergency medical technicians (EMTs) to be 0.6%, well-above the incidence rate of 0.01% for the general public in the same metropolitan area.^7^ As many as 13% of quarantined Toronto paramedics during the 2003 SARS pandemic developed symptoms.^8^

It is reasonable to believe that other FRs involved in direct patient care, such as law enforcement (LE) or firefighters (FFs), also have a higher incidence of COVID-19 infection when compared to the general population. This information has implications both for infection control and for operational decision making. The purpose of the current study was to determine the serological prevalence of past COVID-19 infection amongst a cohort of municipal LE and FFs and to compare it to the local prevalence in the general population.

## Methods

### Study Design

The study is a descriptive analysis of de-identified data reporting serology results for municipal FRs. The study was reviewed by the Mayo Clinic (Rochester, Minnesota USA) Institutional Review Board (IRB 20-005660) and deemed exempt.

### Study Setting

On the weekend of May 16-17, 2020, municipal FRs from the Rochester Police Department and Rochester Fire Department were offered voluntary, anonymous SARS-CoV-2 serology. Blood samples were collected by trained phlebotomists, prepared and transported in accordance with the specific laboratory test requirements.

Results were reported to individual patients, and two follow-up questions were asked:

1. Since February 2020, have you suffered from an illness (eg, fever or chills, cough, shortness of breath or difficulty breathing, headache, sore throat, new loss of taste or smell, congestion or runny nose, nausea or vomiting, or diarrhea) that made you suspect that you might have COVID-19?
2. Have you received a nasal swab for COVID-19? If yes, what was the result?

These results were recorded in a Microsoft Excel database (Microsoft Corporation; Redmond, Washington USA). A de-identified copy of the dataset, containing only the serology results and responses to questions, was made available to the study team for analysis.

### SARS-CoV-2 IgG Serology Test Characteristics

The SARS-CoV-2 IgG serology test (COR2G) was performed by Mayo Clinic Laboratories.^9^ The test is an enzyme-linked immunoassay (ELISA) using a serum sample collected in a serum gel tube and centrifuged. Results are reported as Negative (Index <1.01), Indeterminate (Index ≥1.01 to <1.21), or Positive (Index ≥1.21). Preliminary assay data indicate minimal cross-reactivity with commonly circulating coronavirus strains OC43, 229E, NL63, HKU1.

The COR2G has a specificity of 99.6% and a sensitivity of 88.1% in samples collected >15 days post-symptom-onset or first positive PCR. The COR2G has a sensitivity of 100.0% in hospitalized patients with more than 14 days of symptoms.

### Community Prevalence Estimate

The number of positive reverse transcriptase – PCR (RT-PCR) nasal swab tests reported by county public health at the time of serology testing was used to estimate the community prevalence.^10,11^

### Data Analysis

Descriptive analyses were generated from the de-identified data set. Two-sided Fisher exact tests were used to compare groups, with an alpha level of 0.05 considered statistically significant.

## Results

### Serology Results

Of 318 FRs eligible for testing, a total of 255 (80.2%) underwent voluntary serology testing (Table 1). Amongst LE personnel, 163/207 (78.7%) were tested, compared with 92/111 FF personnel (82.9%; P = .46). The prevalence of positive serology for all FRs tested was 3/255 (1.2%). Two LE (1.2%) and one FF (1.1%) responder had positive serology (P = 1.0; Table 2).

**Table 1.** Law Enforcement and Fire Department Study Participant Demographics.

Agency	Size	Serology Testing, n (%, 95% CI)	Symptoms, n (%, 95% CI)	RT-PCR Testing, n (%, 95% CI)
Law Enforcement	207	163 (78.7%, 72.5% - 84.1%)	44 (27.0%, 20.4% - 34.5%)	5 (3.1%, 1.0% - 7.0%)
Fire Department	111	92 (82.9%, 74.6% - 89.4%)	14 (15.2%, 8.6% - 24.2%)	4 (4.3%, 1.2% - 10.8%)

Note: Subjective report of suffering from an illness since 2/2020 (eg, fever or chills, cough, shortness of breath or difficulty breathing, headache, sore throat, new loss of taste or smell, congestion or runny nose, nausea or vomiting, or diarrhea).

Abbreviation: RT-PCR, reverse transcriptase – polymerase chain reaction.

**Table 2.** Serology and RT-PCR Results of Law Enforcement and Fire Department Study Participants

	**Positive Serology** n (%; 95% CI)	Positive PCR
**Law Enforcement** (n = 163)	2 (1.2%; 0.15% - 4.4%)	0/5
**Firefighters** (n = 92)	1 (1.1%; 0.03% - 5.9%)	0/4

Abbreviation: RT-PCR, reverse transcriptase – polymerase chain reaction.

### COVID-19-Like Symptoms and RT-PCR Testing

Two hundred and twenty-four FRs responded to an institutional survey regarding prior symptoms and testing. Fifty-eight (25.9%) FRs (44 LE, 14 FFs) reported the presence of COVID-19-like symptoms since February 2020 while the remaining 166 (74.1%) were asymptomatic (Table 1). Of the 58 responders reporting COVID-19-like symptoms, nine (15.5%) received RT-PCR testing, of whom none were positive (Table 2).

Two of three FRs with positive serology reported no COVID-19-like symptoms since February 2020, and none of these responders had received prior nasal RT-PCR swabs.

### Community Prevalence Estimate

At the time of FR testing, there had been 575 PCR confirmed COVID-19 cases among the local county population. Based upon United States Census Bureau (Suitland, Maryland USA) county population data, the prevalence of COVID-19 cases in the community was estimated at 0.36%. Thus, FRs demonstrated a three-fold higher positive seroprevalence rate compared with the general population (P = .07).

## Discussion

The current study demonstrated a SARS-CoV-2 IgG Ab prevalence of 1.2% amongst a cohort of municipal FFs and LE, a three-fold higher prevalence compared with the 0.36% estimate amongst the general population. First responders represent a high-risk group due to their constant interface with the public, often times in uncontrolled environments, closed spaces, and with varying patient acuity and cooperativeness.^12^ Previous experience with the 2003 SARS epidemic demonstrated increased probable or confirmed infection rates in EMTs and paramedics in Taiwan and Toronto, respectively, compared to the local population.^7,8^

The FR prevalence in this study, although three-fold higher than the general community, was surprisingly low based upon experience with the previous SARS epidemic. There are several potential explanations for this. Shortly after the recognition of the COVID-19 pandemic in the United States, many municipal agencies changed their approach to medical response calls. This included the use of PPE on all medical calls, the use of a “scout” FR to make initial contact and assessment, thereby limiting unnecessary crew contact and minimization of potential aerosol generating procedures. Strict workplace control measures included enforced social distancing, ready access to hand sanitizers and masks, and changes in shift sign-outs and shift cycles. These changes, in conjunction with a relatively low community prevalence rate, may have served to limit infection amongst FRs.

Another potential explanation for the low FR prevalence may be loss of immunogenicity over time. At this point, the duration of SAR-CoV-2 Ab is unknown. In contrast with SARS, in which immunogenicity lasted for at least one year, preliminary data suggest that immunogenicity to COVID-19 may be lost as rapidly as a few weeks after symptom onset.^13-15^ Lastly, infected individuals may not yet have seroconverted to produce IgG Ab. A recent study documented IgG seroconversion rates of 79.8% at 15 days after disease onset, suggesting that this is not the cause of the low serology rate.^4^

Several additional important findings are highlighted by this study. Although the number of positive serologies was low, two of the three positive FRs reported no signs or symptoms of infection. None of the three positive cases received a RT-PCR-based nasopharyngeal test. This highlights the importance of workplace control measures to prevent infection spread by asymptomatic individuals.

Despite the availability of free serological testing, nearly 20% of eligible FRs did not participate. The reasons for this are unclear and may include lack of availability during the two-day period. However, this may reflect some underlying beliefs about disease risk and susceptibility, which in turn may place departments at risk of inadvertent infection spread.

Approximately one-quarter of all FRs reported the presence of symptoms concerning for possible COVID-19 infection. However, only 15.5% of these individuals received RT-PCR screening for these symptoms. Multiple testing venues existed for these FR, including drive-through testing centers. This would suggest that significant barriers exist in the testing of critical infrastructure personnel. Several individuals reported requesting testing and being denied as they did not meet the then-current Centers for Disease Control and Prevention (CDC; Atlanta, Georgia USA) testing criteria.

## Limitations

This study has several important limitations. Most importantly, the actual prevalence of COVID-19 in the community remains unclear and is estimated upon health department-reported PCR positive cases. The PCR-based testing was frequently performed as a diagnostic study based upon symptoms, rather than as an attempt to determine prevalence. Data from Los Angeles County (California USA) demonstrated a community prevalence of 4.65%, 43.5-fold greater than the number of PCR-confirmed positive cases.^16^ Equally importantly, the estimated prevalence in this community is relatively low compared with national hotspots, impacting FR exposure risk. Although the sensitivity and specificity of the test are high, there remains the potential for both false positive and false negative tests, which would substantially alter the results given the low number of positive serology results. The data also include only 80.2% of the cohort FR population. The study is subject to recall and reporting bias in terms of COVID-19 symptomatology and interpretation of illness. In order to preserve participant anonymity, the study did not collect detailed information regarding participants’ symptomatology, nor did investigators attempt to perform contact tracing among the positive cases.

## Conclusions

Amongst a cohort of municipal FRs, the seroprevalence of SARS-CoV-19 IgG Ab was 1.2%. Two-thirds of positive FRs reported a lack of symptoms. Only 15.5% of FRs with COVID-19-like symptoms received RT-PCR testing. In addition to workplace control measures, increased testing availability to FRs is critical in limiting infection spread and ensuring response capability. Longitudinal surveillance is required to monitor disease incidence over time.
